# Having a usual source of care and its associated factors in Korean adults: a cross-sectional study of the 2012 Korea Health Panel Survey

**DOI:** 10.1186/s12875-016-0555-3

**Published:** 2016-11-29

**Authors:** Ah Reum An, Kyoungwoo Kim, Jae-Ho Lee, Nak-Jin Sung, Sang-il Lee, Min Kyung Hyun

**Affiliations:** 1 Public Health Medical Service Department, Seoul National University Hospital, Seoul, Korea; 2 Department of Family Medicine, Inje University Paik Hospital, Seoul, Korea; 3 Department of Family Medicine, The Catholic University of Korea College of Medicine, 222 Banpo-daero, Seocho-gu, Seoul 137-701 Korea; 4 Department of Family Medicine, Dongguk University Ilsan Hospital, Goyang, Korea; 5 Department of Preventive Medicine, College of Medicine, University of Ulsan, Seoul, Korea; 6 Department of Preventive Medicine, College of Korean Medicine, Dongguk University, Gyeongju, Republic of Korea

**Keywords:** Primary health care, Surveys and questionnaires, Health policy, Korea

## Abstract

**Background:**

Usual source of care (USC) is one of the hallmarks of primary care. We aimed to examine the status of having a USC and its patient-related sociodemographic factors among Korean adults.

**Methods:**

Data were obtained from the 2012 Korea Health Panel survey. Panel participants were selected for the study who were aged 18 years or older and who replied to questionnaire items on having a USC (*n* = 11,935).

**Results:**

Of the participants, 21.5% had a usual place and 13.9% had a usual physician. Reasons for not having a USC were seldom being ill (66.1%), the preference to visit multiple medical institutions (27.9%), and others. The private community clinic was the most common type of usual place (57.0%). In patient-reported attributes of care provided by a usual physician, the percentages of positive responses for comprehensiveness and coordination were 67.2% and 34.5%, respectively. By institution type, primary care clinics showed the lowest percentage (32.8%) of positive responses for coordination. Adjusted odds ratios of having a usual physician were 3.77 (95% confidence interval, CI: 3.75–3.79) for those aged 65 years or older (vs. aged 18–34 years), 1.31 (CI: 1.30–1.31) for females (vs. males), 0.72 (CI: 0.72–0.73) for unmarried people (vs. married), 1.16 (CI: 1.16–1.16) for college graduates or higher (vs. elementary school graduate or less), 0.64 for the fifth quintile (vs. the first quintile) by household income, 1.53 (CI: 1.52–1.54) for Medical Aid (vs. employee health insurance) for type of health insurance, and 4.09 (CI: 4.08–4.10) for presence (vs. absence) of a chronic diseases.

**Conclusions:**

The proportion of Korean adults who have a USC is extremely low, the most influential factor of having a USC is having a chronic disease or not, and Korean patients experience much poorer health care coordination than do patients in other industrialized countries. The findings of this study will give insight to researchers and policy makers regarding the potential facilitators of and barriers to promoting having a USC in the general Korean public.

## Background

The Republic of Korea (Korea) notified the WHO of the first laboratory-confirmed case of the Middle East respiratory syndrome coronavirus (MERS-CoV) on May 20, 2015; the patient was someone who had recently travelled to the Middle East. The index patient transmitted infection to close relatives, patients, and health care workers, and subsequently, some of these patients further infected others in a similar pattern. This was the largest outbreak of MERS outside of the Middle East. The large number of health care facilities involved in this event could be explained by the fact that many patients had visited multiple health care facilities before being isolated, and thus these exposures happened before MERS-CoV was suspected or diagnosed. The practice of seeking care at a number of medical facilities (“doctor shopping”) may have been a contributing factor [1]. Meanwhile, the average number of doctor consultations per person has increased in many OECD countries since 2000. This was particularly the case in Korea, where in 2013 the number of such consultations was the highest (14.6) among the OECD countries compared with the OECD average of 6.5 per person per year [2].

In the background of the MERS outbreak and Korea’s extreme health indicators, there exist structural problems in the country’s health care system, e.g., private-sector dominance in health care institutions, political negligence of primary care, and long-standing fee-for-service payments even though the national universal health insurance system has been in operation since 1989. People usually do not have a primary care physician as a usual source of medical care (USC) such as general practitioners (GPs) in Western industrialized countries [3]. In many countries (e.g., Denmark, the Netherlands, the United Kingdom), GPs address a wide range of patient health problems. When necessary, they refer patients to other health care facilities for further examination or treatment and coordinate the various health care services with outside providers [4]. In Korea, however, people can freely visit medical institutions, see specialists directly on their own, and receive medical treatments by different providers for each episode of care.

A USC is the particular medical professional, doctor's office, clinic, health center, or other place where a person would usually go if sick or in need of advice about his or her health [5]. A USC in this study does not indicate a lay person who has primary responsibility for the day-to-day care of patients. According to previous studies, having a USC is important for quality of care and efficiency in health care and for health itself. Patients who report having a USC report greater trust and satisfaction with their providers [5], are more likely to receive preventive screenings and treatment for chronic health conditions, [6–9], and report fewer unmet service needs [7]. In addition, having a USC could reduce hospitalization costs [10], provide more effective and equitable care [11], increase vaccination coverage [12], enhance timely access to medical care [13], improve quality of care received, and result in improved health status [14]. More foundationally, having a usual physician was more important than having a usual place for receipt of certain preventive services such as blood pressure and cholesterol level checks for adults [14]. A combination of usual place and usual provider is a more coordinated approach to providing preventive care, leading to increased access and better health outcomes, whereas having a place for care but no usual provider, or not having a usual place, are both less advantageous [15]. Studies on having a USC have not been performed in countries with formal patient registration associated with capitation systems. Most of the studies were based on self-reported surveys such as the Medical Expenditure Panel Survey in the United States, and there is little knowledge regarding the current status and influence of having a USC in Korea.

In this study, we aim to provide evidence on the current status of having a physician as a USC, its associated factors, and attributes of care provided by a usual physician, which is expected to be useful when policy makers prepare a plan to reform the Korean health care system.

## Methods

### Data source

The Korea Health Panel (KHP) data are collected for a national database established by a consortium of the Korea Institute for Health and Social Affairs and National Health Insurance Corporation [16]. The KHP uses stratified multistage probability sampling according to region and residence in order to select nationwide subjects from the 2005 Korea Census. The data were initially collected from 7,009 households and 21,283 individuals in 2008, with 5,434 households and 15,872 individuals remaining in 2012. In order to support policies that could correspond to the rapidly changing health care environment with the aging population, medical progress, medical service expansion, and people’s demands for health, the database contains detailed information on families and individuals from a nationally representative sample of households on the following: demographic characteristics, income, savings and expenses, employment, housing, chronic conditions, use of medical services, medications, charges and sources of payments, private health insurance, pregnancy and delivery, elder care, and health behaviors and awareness.

### Variables for having a usual source of care

Questionnaire items regarding having a USC in the KHP survey were asked of adults aged 18 years and over. Items on a place (a medical institution) as a USC were included first in 2009, but items on a physician as a USC were not included before 2012. Three items related to having a usual place: “Do you have a medical institution that you usually visit when you are ill or when you are trying to get a medical check-up or consultation?”, “What is the type of medical institution you usually visit?”, and “What is the reason you do not have a medical institution that you usually visit?” Four items related to having a usual physician: “Do you have a medical doctor who you usually see when you are ill or when you are trying to get a medical check-up or consultation? (first-contact)”, “How long has it been since you first saw the medical doctor? (longitudinal relationship)” “Does the medical doctor solve almost all the common health problems that you have? (comprehensiveness)” “Does the medical doctor appropriately introduce you to health care facilities and providers for your health? (coordination)?” The items on comprehensiveness and coordination are rated on 5-point Likert scales.

First, we analyzed the percentage of having a USC among adult panel participants aged 18 and over by institution type and sociodemographic variables. When panel participants had a USC, we addressed the two items of whether the USC was a place or both a place and a physician to compare the characteristics of both choices.

### Statistical analysis

Having a USC (place or physician plus place) or not was analyzed descriptively to show the distributions by institution type and sociodemographic variables: age, sex, marital status, household income by quintile, education (years), type of health insurance (employee, self-employment, Medical Aid), chronic disease, experience of admission and emergency room visit during the past year.

Multiple logistic regressions were performed to identify the sociodemographic factors (including chronic disease in Model 2 but not Model 1) that were associated with having a physician as a USC. The Hosmer-Lemeshow test [17] was applied to test the goodness of fit of the logistic regression models. The models’ discriminative ability was assessed using the concordance statistic, a unit-less index that denotes the probability that a randomly selected subject who experienced the event will have a higher predicted probability of having the outcome occur compared with a randomly selected subject who did not experience the event [18].

Statistical analyses were performed with SAS version 9.4 (SAS Institute, Cary, North Carolina, USA) software, with *P* = 0.05 or less regarded as a significant difference.

## Results

### Baseline characteristics and status of having a USC

There were 11,946 adults aged 18 or older in the 2012 Korea Health Panel. Eleven did not respond to questions about having a USC, so the interview responses of 11,935 adults were analyzed. Among those who had a USC, 21.5% had a place (institution with or without a physician) and 13.9% had a physician (Table 1).

**Table 1:** Distributions of sociodemographic variables by the type of usual source of care in the 2012 Korean Health Panel

		Place as a USC			Physician as a USC			
		Have (%)	Does not have (%)		Have (%)	Does not have (%)		Total
		2,918 (21.5)	9,017 (78.5)	*P*	1,909 (13.9)	10,026 (86.1)	*P*	11,935 (100)
Age	18–34	174 (7.8)	2,180 (92.2)	<0.001	87 (3.8)	2,267 (96.2)	<0.001	2,354 (100)
	35–49	575 (15.9)	3,051 (84.1)		386 (10.7)	3,240 (89.3)		3,626 (100)
	50–64	889 (28.7)	2,156 (71.4)		590 (18.7)	2,455 (81.3)		3,045 (100)
	65 –	1,280 (44.6)	1,630 (55.4)		846 (30.0)	2,064 (70.0)		2,910 (100)
Sex	Male	1,199 (18.3)	4,380 (81.7)	<0.001	771 (11.5)	4,808 (88.5)	<0.001	5,579 (100)
	Female	1,719 (24.5)	4,637 (75.5)		1,138 (16.2)	5,218 (83.8)		6,356 (100)
Marital status	Married	2,229 (24.5)	6,064 (75.5)	<0.001	1,471 (16.0)	6,822 (84,0)	<0.001	8,293 (100)
	Divorced/separated/widowed	525 (34.8)	926 (65.2)		349 (23.8)	1,102 (76.2)		1,451 (100)
	Unmarried	164 (7.5)	2,027 (92.5)		89 (4.1)	2,102 (95.9)		2,191 (100)
Education (unit: year)	0–6	1,014 (39.2)	1,535 (60.8)	<0.001	664 (26.2)	1,885 (73.8)	<0.001	2,549 (100)
	7–12	1,281 (23.2)	3,811 (76.8)		861 (15.5)	4,231 (84.5)		5,092 (100)
	13 –	623 (13.5)	3,671 (86.5)		384 (8.0)	3,910 (92.0)		4,294 (100)
Household income (unit: quintile) (missing = 13)	First (lowest)	739 (39.2)	1,033 (60.8)	<0.001	499 (25.6)	1,273 (73.4)	<0.001	1,772 (100)
	Second	553 (21.9)	1,711 (78.1)		378 (15.5)	1,886 (84.5)		2,264 (100)
	Third	582 (19.9)	1,973 (80.1)		388 (13.3)	2,167 (86.7)		2,555 (100)
	Fourth	532 (19.1)	2,094 (80.9)		327 (11.7)	2,299 (88.3)		2,626 (100)
	Fifth (highest)	509 (17.1)	2,196 (82.9)		314 (10.0)	2,391 (90.0)		2,705 (100)
Type of health insurance (missing = 64)	Employee	1,857 (20.4)	6,085 (79.6)	<0.001	1,193 (12.8)	6,749 (87.2)	<0.001	7,942 (100)
	Self-employment	806 (21.3)	2,623 (78.7)		547 (14.6)	2,882 (85.4)		3,429 (100)
	Medical Aid	221 (41.3)	279 (58.7)		153 (28.8)	347 (71.2)		500 (100)
Chronic disease	Presence	2,576 (33.1)	4,737 (66.9)	<0.001	1,715 (22.1)	5,598 (77.9)	<0.001	7,313 (100)
	Absence	342 (7.3)	4,280 (92.7)		194 (4.0)	4,428 (96.0)		4,622 (100)

Chi-square test. USC, usual source of care. Population-based cross-sectional weights were applied to percentages

Unadjusted analysis showed that distributions of those who had a USC were significantly different by all sociodemographic variables (*P* < 0.001). The percentages of having a USC were higher with age (of age 65 or older, 44.6% have a place and 30.0% have a physician); with female sex (24.5% have a place and 16.2% have a physician); with marital status divorced or separated or widowed (34.8% have a place and 23.8% have a physician); with less education (of six years or less, 39.2% have a place and 26.2% have a physician); by lower household income by quintile (in the 1^st^ quintile, 39.2% have a place and 25.6% have a physician); with Medical Aid as health insurance was Medical Aid (41.3% have a place and 28.8% have a physician); and with presence of a chronic disease (33.1% have a place and 22.1% have a physician; see Table 1).

The most frequent reason for not having a USC was seldom being ill (66.1%), followed by preferring to visit multiple medical institutions (27.9%) (Table 2).

**Table 2:** Reasons for not having a place as a usual source of care on the 2012 Korean Health Panel

Seldom ill	5,493 (66.1)
Prefer to visit multiple medical institutions	2,956 (27.9)
Prefer to take care of oneself and rarely use a medical institution	275 (2.9)
Don’t know where to go	196 (2.1)
Others	95 (1.0)
Non-responders	2 (0.0)
Total	9,017 (100)

Population-based cross-sectional weights were applied to percentages

By type of institution, the most common place as a USC was a private community clinic (57.0%), followed by a community hospital (20.9%). The distributions of those who had physicians as a USC by type of institution of a USC were significantly different (*P* < 0.001); those who had a combination of a place and a physician as a USC cited private community clinics as their USCs more frequently than did those who had only a place without a physician as a USC (61.8% vs. 48.6%); for hospitals, though, the findings were the reverse (Table 3).

**Table 3:** Distributions of usual places of care by having or not having a physician as a USC among those who have a place as a USC on the 2012 Korea Health Panel

Usual places		Physician as a USC		
	Total (%)	Have (%)	Does not have (%)	*P*
Public community clinics	132 (4.0)	74 (3.6)	58 (4.7)	
Private community clinics	1,685 (57.0)	1,151 (61.8)	534 (48.6)	
Community hospitals	582 (20.9)	322 (16.9)	260 (27.9)	<0.001
General/University Hospitals	491 (17.1)	314 (16.8)	177 (17.6)	
Sites for traditional medicine	26 (0.9)	14 (0.9)	12 (1.0)	
Others	2 (0.1)	0	2 (0.2)	
Total^a^	2,918 (100)	1,875 (100)	1,043 (100)	

Chi-square test. USC, usual source of care. Population-based cross-sectional weights were applied to percentages

^a^The number of those who had a usual physician without a usual place (34) was excluded

### Patient-reported attributes of care served by a physician as a USC

Regarding comprehensiveness, 67.1% of the panelists who had a particular physician as a usual source of care agreed that they resolved most of their health problems with their physicians’ care. By type of institution, 66.7% of people whose physicians were in primary care clinics, 70.0% in community hospitals, and 66.2% in general or university hospitals answered that they experienced good or very good comprehensiveness at all facilities. People who had a regular physician in a community hospital cited the highest proportion of positive experiences with comprehensiveness of care (*P* < 0.001). Regarding coordination, 34.5% of people who had a usual physician of care agreed that their physicians referred them properly to other health care facilities or providers. By type of institution, 32.8% of people whose regular physicians were in primary care clinics, 40.2% in community hospitals, and 35.5% in general or university hospitals answered that their care coordination was good or very good. The assessments of coordination in primary care clinic were lowest (*P* < 0.001).

Regarding longitudinality, the median duration ± interquartile range of a doctor-patient relationship was 5.0 ± 7.0 years, and there were no significant differences between the types of institutions the regular physicians worked for (*P* = 0.077; Table 4).

**Table 4:** Attributes of care provided by a physician as a usual source of care (USC) by type of institution on the 2012 Korea Health Panel

Attributes of care provided by a physician as a USC		Types of institution of physicians as a USC				
		Total	Primary care clinic	Community hospital	General or university hospital	
		1,861 (100)	1,225 (100)	322 (100)	314 (100)	*P*
Comprehensiveness; frequency (%)	Very good	162 (8.7)	110 (9.0)	25 (8.2)	27 (8.0)	<0.001
	Good	1,093 (58.4)	703 (57.7)	206 (61.8)	184 (58.2)	
	Fair	362 (19.5)	250 (19.8)	51 (17.5)	61 (20.3)	
	Poor	154 (8.3)	106 (8.7)	21 (6.1)	27 (8.9)	
	Very poor	90 (5.1)	56 (4.8)	19 (6.5)	15 (4.6)	
Coordination; frequency (%)	Very good	89 (5.4)	52 (4.9)	23 (8.3)	14 (4.8)	<0.001
	Good	541 (29.1)	331 (27.9)	113 (31.9)	97 (30.7)	
	Fair	435 (23.1)	281 (22.7)	76 (24.4)	78 (23.1)	
	Poor	343 (17.7)	240 (18.4)	50 (15.7)	53 (17.2)	
	Very poor	453 (24.7)	321 (26.1)	60 (19.7)	72 (24.2)	
Longitudinal duration of physician-patient relationship (year) (median ± IQR)		5.0 ± 7.0	6.0 ± 7.0	5.0 ± 7.0	5.0 ± 7.0	0.077

Chi-square test & Kruskal-Wallis test. USC, usual source of care; IQR, interquartile range. Population-based cross-sectional weights were applied to percentages. Primary care clinics include public and private community clinics. Those who had a physician without a place (*n* = 34), an institution for traditional medicine (*n* = 26), and others (*n* = 2) as a USC were excluded

### Sociodemographic factors associated with having a physician as a USC

In multiple logistic regressions to find the factors associated with having a regular physician, we considered two models according to whether having or not having a chronic disease, because not being ill was the most frequent reason for not having a USC as noted in the Table 2. As independent variables, age, sex, marital status, education level, household income, and type of health insurance were included in Model 1, and chronic disease added to Model 2. Model 2 was found to be more appropriate than Model 1 (*P* = 0.653 vs. 0.163 with Hosmer-Lemeshow goodness of fit test; 0.735 vs. 0.705 with the concordance statistics).

In regression Model 2, all independent variables were significantly associated with having a USC. Adjusted odds ratios (ORs) of having a physician as a USC were 3.77 (95% confidence interval (CI): 3.75–3.79) for being aged 65 years or older (vs. 18–34 years), 1.31 (CI: 1.30–1.31) for being female (vs. male), 0.72 (CI: 0.72–0.73) for being unmarried (vs. married), 1.16 (CI: 1.16–1.16) for 13 years or more (vs. 6 years or less) of education, 0.64 (CI: 0.64–0.64) for income in the fifth (vs. the first) quintile, 1.53 (CI: 1.52–1.54) for having Medical Aid (vs. employee health insurance), and 4.09 (CI: 4.08–4.10) for the presence (vs. absence) of a chronic disease (Table 5).

**Table 5:** Sociodemographic factors associated with having a physician as a usual source of care on the 2012 Korean Health Panel

		Model 1		Model 2	
		Odds ratio	95% CI	Odds ratio	95% CI
Age (year)	18–34	1		1	
	35–49	2.23	2.22–2.24	1.85	1.85–1.86
	50–64	4.15	4.13–4.17	2.60	2.59–2.62
	65 –	6.92	6.89–6.96	3.77	3.75–3.79
Sex	Male	1		1	
	Female	1.42	1.42–1.42	1.31	1.31–1.31
Marital status	Married	1		1	
	Divorced/separated/widowed	0.83	0.82–0.83	0.84	0.84–0.84
	Unmarried	0.63	0.62–0.63	0.72	0.72–0.73
Education (year)	0–6	1		1	
	7–12	1.09	1.08–1.09	1.12	1.12–1.13
	13 –	1.03	1.03–1.03	1.16	1.16–1.16
Household income (quintile)	First (lowest)	1		1	
	Second	0.79	0.79–0.79	0.79	0.79–0.80
	Third	0.79	0.79–0.80	0.82	0.82–0.83
	Fourth	0.75	0.75–0.75	0.77	0.77–0.77
	Fifth (highest)	0.65	0.65–0.65	0.64	0.64–0.64
Type of health insurance	Employee	1		1	
	Self-employment	1.06	1.06–1.06	1.09	1.08–1.09
	Medical Aid	1.73	1.72–1.74	1.53	1.52–1.54
Chronic disease	Absence			1	
	Presence			4.09	4.08–4.10
*P* values for the Hosmer-Lemeshow tests of model fit		0.163		0.656	
Concordance statistic for discriminative ability		0.705		0.735	
Pseudo R-square		0.092		0.128	

Multiple logistic regression analysis

*CI* confidence interval

## Discussion

Primary care is the backbone of national health care systems [11, 19]. The positive impact of primary care on health comes from comparing the health of people who do or do not have a primary care physician as their USC [19]. This study disclosed fragile aspects of the Korean health care system by demonstrating that only 13.9% of adults in the 2012 KHP, a nationally representative sample, have a physician as a USC (21.5% have a place as a USC). A 2007 international survey by the Commonwealth Fund that consisted of interviews with representative samples of adults age 18 and older in seven countries found that the proportions of people who had a USC (one doctor and only one place) in other countries was 100% (100% and 0%) in the Netherlands, 97% (89% and 8%) in the United Kingdom, 96% (88% and 8%) in Australia, 95% (92% and 3%) in Germany, 95% (89% and 6%) in New Zealand, 91% (84% and 7%) in Canada, and 90% (80% and 10%) in the United States [20]. In addition, this study showed that only 34.5% answered positively regarding the coordination of care they experienced from their usual physicians among those who had a usual physician in the 2012 KHP. This finding is another extreme index of primary care in Korea that contrasts explicitly with data from other industrialized countries. In the above international survey, the proportions of those reported receiving coordinated care always or often were 70% in Australia, 69% in the United States, 67% in Canada and Germany, 60% in New Zealand, 58% in the United Kingdom, and 55% in the Netherlands [20].

This low proportion of having a USC in Korea is related to the weak primary health care system [21]. Korea achieved universal health insurance coverage in 1989 and integrated it into a single insurer, the National Health Insurance Program, with no patient-list system in primary care [22]. Health care providers obligatorily made contracts with the national health insurance corporation [23], but people can visit any health care provider without referrals from their primary care physicians. Having health insurance is important for having a USC [24]. However, the low proportion of Korean adults who have a USC means that health insurance alone is not sufficient for having a USC and that Korea requires health policies that promote having a USC.

The most common reason on the 2012 KHP for not having a USC was “seldom being ill” (66.1%). People who needed medical care seemed more likely to have a USC than did people who did not need medical care. According to the Medical Expenditure Panel Survey in the United States, 63.0% individuals without a USC answered “Seldom or never get sick” as their main reason for not having a USC [25], which was similar to our results. However, the second-most common reason for not having a USC differed between the two countries: In Korea, it was “the preference to visit multiple medical institutions” (27.9%), whereas in the United States, it was the cost of medical care (14%) [25]. The fact that there is a significant portion of people who prefer visiting multiple places (any kind of medical specialists) implies that voluntary participation, rather than mandatory registration, could be an important manner to disseminate having a USC without resistance of the public in Korea.

Thirty-eight percent of the people who had a USC considered a community, general, or university hospital to be their usual place of care; these patients were likely to have chronic conditions, and patients with severe conditions need hospital care more than clinic care. Among the medical institutions for which usual physicians worked, the proportion of hospitals was 33.7% in this study. Because hospital ambulatory care can be easily accessed in Korea, a usual hospital physician who can provide multi-morbid patients with coordinated care may be a pragmatic alternative USC from the patients’ perspective. In reality, however, care coordination in Korea is very poor, and actual health outcomes according to the types of USC are rarely studied. Previous studies in Western countries found that adults who reported having a primary care physician rather than a specialist as a USC had lower subsequent five-year mortality rates after initial differences in health status, demographic characteristics, health insurance status, health perceptions, reported diagnoses, and smoking status were controlled for [26]. Further research is needed to reveal the preferred type of USCs for improving health outcomes in Korean adults.

It is evident that improving primary care in Korea is critical to ensuring care quality [21]. People should receive comprehensive and coordinated care by the first-contact physician in their own community. However, in this study, the patients’ assessments of care comprehensiveness and coordination provided by their USCs were even poorer for community private clinics. The main reason is that primary care in Korea has structural and functional weaknesses: the overwhelming superiority of solo practices, lack of multidisciplinary team approaches, long-standing exclusive fee-for-service payments with low fees and limited benefits for patient education or counseling, and lack of sharing patient health information between clinics and hospitals. In essence, there is a lack of consensus on the roles of standard primary care in care comprehensiveness and coordination.

This study does have some limitations. First, it could not address the discrete characteristics of primary care in Korea (e.g., primary care practices and physicians) because the KHP survey questionnaire did not include those items even though having a USC is a basic component of longitudinality in primary care. Second, the authors of this study analyzed the cross-sectional data from 2012 only because the items regarding a usual physician had not been included in the questionnaire before 2012, although panel data did improve opportunities to describe growth and development trajectories over the life course and to study the patterns of causal relationships over longer time spans between having a USC and health outcomes.

## Conclusions

In conclusion, the proportion of Korean adults who have a USC is extremely low (21.5% for place and 13.9% for physician), the most influential factor of having a USC is having a chronic disease, and Korean patients experience much poorer health care coordination than do patients in other industrialized countries [21]. This fact is related to the weak Korean primary care system and can threaten the system’s quality and efficiency, leading to unnecessary costs. The findings of this study will give insight to researchers and policy makers regarding the potential facilitators of and barriers to promoting having a USC among the general Korean public.

## Abbreviations

CI: Confidence interval
GP: General practitioners
KHP: Korea Health Panel
MERS-CoV: Middle East respiratory syndrome coronavirus
OECD: Economic Cooperation and Development
OR: Odds ratio
USC: Usual source of care
WHO: World Health Organization

## References

[1] WHO. Middle East respiratory syndrome coronavirus (MERS-CoV): summary and risk assessment of current situation in the Republic of Korea and China as of 19 June 2015. Geneva: WHO. Available at: http://www.who.int/emergencies/mers-cov/mers-cov-republic-of-korea-and-china-risk-assessment-19-june-2015.pdf. Accessed 6 Jun 2016.

[2] OECD (2015). Health at a Glance 2015: OECD Indicators.

[3] Chun CB, Kim SY, Lee JY, Lee SY (2009). Republic of Korea: Health system review. Health Syst Transit.

[4] Mossialos E, Wenzl M, Osborn R, Sarnak D, editors. International profiles of health care systems. 2015. The Commonwealth Fund, January 2016. Available at: http://www.commonwealthfund.org/~/media/files/publications/fund-report/2016/jan/1857_mossialos_intl_profiles_2015_v7.pdf. Accessed 6 Jun 2016.

[5] Carpenter WR, Godley PA, Clark JA, Talcott JA, Finnegan T, Mishel M, Bensen J, Rayford W, Su LJ, Fontham ET, Mohler JL (2009). Racial differences in trust and regular source of patient care and the implications for prostate cancer screening use. Cancer.

[6] Kim MY, Kim JH, Choi IK, Hwang IH, Kim SY (2012). Effects of having a usual source of care on preventive services and chronic disease control: a systematic review. Korean J Fam Med.

[7] DeVoe JE, Tillotson CJ, Wallace LS (2009). Usual source of care as a health insurance substitute for U.S. adults with diabetes?. Diabetes Care.

[8] DeVoe JE, Tillotson CJ, Lesko SE, Wallace LS, Angier H (2011). The case for synergy between a usual source of care and health insurance coverage. J Gen Intern Med.

[9] Ahmed NU, Pelletier V, Winter K, Albatineh AN (2013). Factors explaining racial/ethnic disparities in rates of physician recommendation for colorectal cancer screening. Am J Public Health.

[10] Damiano PC, Momany ET, Tyler MC, Penziner AJ, Lobas JG (2006). Cost of outpatient medical care for children and youth with special health care needs: investigating the impact of the medical home. Pediatrics.

[11] Starfield B, Shi L (2004). The medical home, access to care, and insurance: a review of evidence. Pediatrics.

[12] Smith PJ, Santoli JM, Chu SY, Ochoa DQ, Rodewald LE (2005). The association between having a medical home and vaccination coverage among children eligible for the vaccines for children program. Pediatrics.

[13] Bartman BA, Moy E, D’Angelo LJ (1997). Access to ambulatory care for adolescents: the role of a usual source of care. J Health Care Poor Underserved.

[14] Xu KT (2002). Usual source of care in preventive service use: a regular doctor versus a regular site. Health Serv Res.

[15] Blewett LA, Johnson PJ, Lee B, Scal PB (2008). When a usual source of care and usual provider matter: adult prevention and screening services. J Gen Intern Med.

[16] Korea Health Panel. About Korea Health Panel. Available at: https://www.khp.re.kr:444/english/about_01.jsp. Accessed 6 Jun 2016.

[17] Hosmer Jr DW, Lemeshow S, Sturdivant RX. Applied logistic regression. John Wiley & Sons 2013.

[18] Austin PC, Steyerberg EW (2012). Interpreting the concordance statistic of a logistic regression model: relation to the variance and odds ratio of a continuous explanatory variable. BMC Med Res Methodol.

[19] Starfield B, Shi L, Macinko J (2005). Contribution of primary care to health systems and health. The Milbank Q.

[20] Schoen C, Osborn R, Doty MM, Bishop M, Peugh J, Murukutla N (2007). Toward higher-performance health systems: adults’ health care experiences in seven countries, 2007. Health Aff (Millwood).

[21] OECD (2012). Raising Standards.

[22] Song YJ (2009). The South Korean health care system. JMAJ.

[23] Kwon S (2009). Thirty years of national health insurance in South Korea: lessons for achieving universal health care coverage. Health Policy Plan.

[24] Newacheck PW, Stoddard JJ, Hughes DC, Pearl M (1998). Health insurance and access to primary care for children. N Engl J Med.

[25] Kirby JB. Main Reason for Not Having a Usual Source of Care by Race, Ethnicity, Income, and Insurance Status, 2007. December 2010. Rockville: Agency for Healthcare Research and Quality; 2007. Available at: https://meps.ahrq.gov/data_files/publications/st308/stat308.shtml. Accessed 6 Jun 2016.

[26] Franks P, Fiscella K (1998). Primary care physicians and specialists as personal physicians. Health care expenditures and mortality experience. J Fam Pract.

